# Patient-Specific iPSC-Derived RPE for Modeling of Retinal Diseases

**DOI:** 10.3390/jcm4040567

**Published:** 2015-03-31

**Authors:** Huy V. Nguyen, Yao Li, Stephen H. Tsang

**Affiliations:** 1 College of Physicians and Surgeons, Columbia University, 100 Haven Ave, Apt 14B, New York, NY 10032, USA; 2 Department of Ophthalmology, Columbia University, 635 W 165th St, New York, NY 10032, USA; E-Mails: yl2635@columbia.edu (Y.L.); sht2@columbia.edu (S.H.T.)

**Keywords:** retinal disease, induced pluripotent stem cells, gene therapy

## Abstract

Inherited retinal diseases, such as age-related macular degeneration and retinitis pigmentosa, are the leading cause of blindness in the developed world. Currently, treatments for these conditions are limited. Recently, considerable attention has been given to the possibility of using patient-specific induced pluripotent stem cells (iPSCs) as a treatment for these conditions. iPSCs reprogrammed from adult somatic cells offer the possibility of generating patient-specific cell lines *in vitro*. In this review, we will discuss the current literature pertaining to iPSC modeling of retinal disease, gene therapy of iPSC-derived retinal pigmented epithelium (RPE) cells, and retinal transplantation. We will focus on the use of iPSCs created from patients with inherited eye diseases for testing the efficacy of gene or drug-based therapies, elucidating previously unknown mechanisms and pathways of disease, and as a source of autologous cells for cell replacement.

## 1. Introduction

Human vision is vital for nearly every major activity of daily living, and degeneration of one of the responsible cell types, the retinal pigmented epithelium (RPE), leads to severe visual impairment and blindness. RPE cells exist as a monolayer located at the back of the eye between the retina and Bruch’s membrane and is essential for photoreceptor function and survival. Retinal diseases such as age-related macular degeneration (AMD) and retinitis pigmentosa (RP) result in clinical pathophysiology characterized by progressive loss of RPE. The adult retina does not intrinsically regenerate, so RPE degeneration may ultimately lead to blindness. Anti-vascular endothelial growth factor (VEGF) therapy has been shown to slow the rate of vision loss, but it has no more than a 10% rate of effectiveness in all AMD cases [1]. There are no other treatments currently established for RPE degenerative diseases, so the disease burden of these conditions are expected to continue to rise. AMD and RP are both leading causes of blindness in the developed world, affecting up to one third of people over the age of 75. Among the elderly, blindness is feared more than any other illness outside of cancer. Currently, nine million Americans have been diagnosed with AMD, and its incidence is expected to double within a decade, affecting 20% of Americans between the ages of 65 and 75 years [2].

Cell transplantation into the human retina has the potential to restore vision and provide treatment in diseases like AMD and RP with significant RPE loss. Since these diseases spare the inner retina and optic nerve, retinal transplantation has focused on replacement of the photoreceptors and RPE. Retinal stem cells have been shown to be efficient at integrating into the degenerative host retina [3]. Replacement of damaged RPE in patients with AMD is now being offered [4]. In 2011, the U.S. Food and Drug Administration advanced the treatment of macular degenerations by approving clinical trials using embryonic stem (ES) cell-derived RPE transplants [5].

Induced pluripotent stem (iPS) cells reprogrammed from adult somatic cells offer the possibility of generating patient-specific cell lines *in vitro*. As a platform to study patient-specific targeted disease cells, iPS cells (iPSC) have exciting potential in regenerative medicine and human disease modeling. As one example, after human embryonic stem cells were shown to be able to produce 3-D optic vesicle-like structures displaying a precise apical-basal orientation [6], human iPS cells were used to also create optic vesicle-like structures which self-assembled into rudimentary, multilayered retinal tissue [7]. Similarly, human iPSCs have been used to model primary open-angle glaucoma (POAG). The optineurin E50K mutation is a mutation currently affirmed as causative for POAG, and human iPSCs have been created with the E50K mutation to study the molecular and cellular characterization of POAG onset [8]. hiPSC modeling has also suggested that normal-tension glaucoma via TBK1 gene duplication is due increased levels of LC3-II, a key marker of autophagy [9].

Specifically, iPS-based therapies holds great promise for treating retinal degenerative diseases, given the advantage of ocular immune privilege and the ease of ocular non-invasive imaging. Moreover, iPS cell technology facilitates investigations of pathophysiological mechanisms of genetic mutations and testing of gene therapy vectors on RPE-based disease models. Indeed, iPS-derived RPE (iPS-RPE) can be reproducibly isolated and closely monitored both morphologically and functionally before experiments, effectively minimizing variability in the timing of differentiation. In addition, RPE, unlike many other human cell types, has a well-described culture standard, which ensures proper controls [4,10].

The *in vitro* phenotypes of disease-specific iPS-derived cells can be used to bridge the gap between the clinical phenotype and molecular or cellular mechanisms, creating new strategies for drug screening, and developing novel therapeutic agents [11]. Human iPS cell-based disease models can prove that a disease is caused by a genetic mutation, hypothesize potential treatment options before using more expensive animal models [12], and assist in the development of novel treatments for clinical trials [13,14,15].

## 2. iPSC Disease Modeling

### 2.1. Use of iPS-Derived RPE Cells for Cell Therapy

The eye is an ideal site for stem cell therapies. First, it is considered an immune privileged organ since the inflammatory responses of the eye differ significantly from those in other tissues. Second, the eye allows for easy accessibility for monitoring and imaging. Third, in the case of serious complications, the eye as a unit can be removed, due to its relative isolation from other body systems. Stem cells in turn are an appealing option for retinal cell replacement due to their pluripotency and potentially unlimited capacity for self-renewal. Currently, there are two leading options for stem cells in retinal transplantation: (i) embryonic stem cells (ESCs), which can be isolated from developing embryos four to five days after fertilization; and (ii) induced pluripotent stem cells (iPSCs), which can be created from adult cells by the viral transduction of transcription factors [16]. However, due to the ethical and technical concerns with using ESCs, iPSCs have largely been favored for retinal transplantation.

iPSCs in particular offer a compelling alternative approach for stem cell therapy. When derived from the transplant recipient, autologous iPS-derived cells reduce the risk of post-transplant rejection and obviate the need for immunosuppression after transplantation. The well-described iPSC culture standards also aid in the development of functional testing and optimization studies. Likewise, RPE transplantation into the retina poses fewer challenges than other kinds of cell transplantation since routine culture of RPE cells has been well described [17,18]. RPE monolayers exist in an easily identifiable hexagonal structure and can be isolated and transferred to a variety of substrates without the need for synaptic integration. Subsequently, studies on RPE replacement therapies using pluripotent stem cells have progressed rapidly. A multicenter trial focusing on the treatment of dry macular degeneration and Stargardt macular dystrophy showed that purified human ESC-derived RPE can be subretinally injected into patients with good results [5]. This is also possible since the retina normally enjoys relative immune privilege, due to the blood-retinal barrier. This barrier consists of non-fenestrated retinal vasculature ensheathed by pericyte and astrocyte processes on the inner aspect and by tight junctions between RPE on the outer aspect. In a healthy state, this blood-retinal barrier provides protection to transplanted cells beneath the retina from the systemic immune system. However, in a diseased RPE state, the monolayer is disrupted due to faulty tight junctions and the retina may also become much more pro-inflammatory [19,20]. Therefore, cells transplanted into a diseased retina are likely to be at a higher risk for rejection, so autologous iPSC transplantation represents the best stem cell approach for curing degenerative retinal diseases. In fact, hiPSC-derived RPE has recently been approved in Japan for use in patient safety trials for treatment of AMD [21].

Currently, human iPS-derived RPE (iPS-RPE) experiments are largely confined to animal models. In 2009, Carr *et al.* performed subretinal injections of dissociated human iPS-RPE into Royal College of Surgeons (RCS) rats and observed restoration of RPE phagocytotic function, as measured by intracellular RHO staining, and long-term preservation of visual function, as measured by optokinetic head-tracking [22]. Another model is the RPE-specific protein 65 kDA (RPE65) mutant mouse model, which is used to study Leber congenital amaurosis (LCA) and RP since the RPE 65 defect leads to a faulty isomerase which can no longer convert the chromophore necessary for rhodopsin to detect light [23]. In 2012, Li *et al.* injected dissociated human iPS-RPE into the subretinal space of the RPE65 mutant mouse model and showed integration of the transplant with host RPE, as well as a modest improvement of visual function as measured by electroretinogram (ERG) [10]. The *Mfrp^rd6^*/*Mfrp^rd6^* (rd6) mouse, which has a deletion in the Membrane Frizzled-Related Protein (*Mfrp)* gene, is another widely used model. The resulting MFRP protein, an RPE-specific membrane receptor of unknown function, is abnormal and the mice exhibit progressive retinal degeneration, making the model a preclinical and progressive model of RP [24]. In a recent study, subretinal injections of AAV-packaged wild-type *Mfrp* into rd6 mice showed improvement in visual function and RPE cell layer thickness [25].

The most advantageous aspect of iPSC based therapy is the potential of autologous transplantation, which intends to address the problem of immune rejection. Despite the assumption that these autologous cells should not provoke an immune response in the recipient from whom the cells were derived, there have been conflicting reports that raise some concern of the immunogenicity of iPSCs. In a recent study, teratomas originating from subcutaneous injection of murine derived iPSCs were found to have abnormal gene expression in some cells, which elicited a T-cell dependent immune response in syngeneic mice [26]. However, when Guha *et al.* transplanted various types of murine iPS-derived cells to a site under the kidney capsule of B6 mice, they found no evidence of immune response to the iPSCs, no increased T cell proliferation *in vitro*, no rejection of syngeneic iPSC-derived cells after transplantation, and no antigen-specific secondary immune response [27]. Findings by Liu *et al.* in 2013 suggests that iPSC immunogenicity increases with *in vivo* differentiation, as the authors observed immune responses after transplantation of differentiated iPS-derived cardiomyocytes but no response when transplanting undifferentiated iPSCs [28]. In contrast, Morizane *et al.* performed a direct comparison between autologous and allogeneic transplantation of iPS-derived neural cells in brains of non-human primates and found that the autologous transplantation of iPS-derived neurons caused only a minimal immune response in the brain, while the allografts elicited an acquired immune response [29]. Moreover, a higher number of dopaminergic neurons survived in autografted iPS-derived cells, which further support their use. Taken together, these findings reveals that different cell types derived from iPSCs might have distinctive immunogenicities in their syngeneic hosts. For the development of human iPS-based cell therapy, there remains still a challenge to evaluate the immunogenicity of human iPS-derived cells in an autologous human immune system.

### 2.2. Progress of RPE Disease Modeling Using iPSCs

Human iPS cells are useful for modeling RPE disorders since they can be isolated, expanded, re-seeded, and closely monitored both morphologically and functionally prior to testing [30]. Phenotypes of patient-specific iPS cells may differ from those from a mouse model with the same mutation [25], underscoring the necessity for multiple models of human genetic diseases. Since differences in phenotypic expression can be observed among species with the same genetic mutation, it is important to study patient-specific cell lines as a complement to mouse models.

The first retinal disease modeled with patient-specific iPS cells is Best vitelliform macular dystrophy (BVMD) [13]. Caused by a defect in the RPE gene BEST1, which results in the subretinal accumulation of photoreceptor waste products, BVMD is characterized by central vision loss due to photoreceptor death. Singh *et al.* created iPS-RPE from affected patients and compared them with those created from unaffected siblings. From their model, they concluded that the pathophysiology of the disease included delayed rhodopsin degradation after photoreceptor outer segment feeding, as evidenced by disrupted fluid flux and increased accumulation of autofluorescent material [13]. This hiPSC model of BVMD possessed functional deficiencies consistent with the clinical features of the disease and was used to characterize clinically relevant disease phenotypes for BVMD.

iPS-derived RPE cells have also recently been used to model and study the pathophysiology of AMD. While genome-wide association studies (GWAS) have identified risk alleles for the disease, such as the ARMS2 and HTRA1 genes, how these alleles lead to pathology is still unclear. There is currently a lack of appropriate models for AMD; autopsy eyes from end-stage patients already possess terminal changes and cannot be used to determine how abnormal gene expression can lead to RPE pathology, and mice do not have maculae. To bypass these obstacles, Yang *et al.* created a model for AMD by obtaining patient-specific iPS-derived RPE and pharmacologically accelerating the aging process with treatment of bisretinoid N-retinylidine-N-ethanolamine (A2E) and blue light [12]. From a proteome screen of multiple A2E-aged patient-specific iPS-RPE lines, impaired superoxide dismutase 2 (SOD2) function was identified as a high risk factor for developing AMD. Using their iPS model, the researchers concluded that the ARMS2/HTRA1 risk alleles decreased SOD2 defense, making RPE more susceptible to oxidative damage and thus contributing to AMD pathogenesis.

## 3. Personalized Medicine: Patient-Specific iPSC-Based Therapy

### 3.1. Development of Gene Therapy on Patient-Specific iPSCs

Gene-corrected patient specific iPSCs offer a unique approach to autologous therapies, which have the potential to treat a wide range of acquired and inherited diseases. However, gene targeting in human pluripotent stem cells has been exceedingly difficult [31]. One approach is using recombinant adeno-associated virus (AAV) as a gene transfer vector to carry the missing gene into affected cells. Vasireddy *et al.* published the first study which successfully transduced iPSCs developed from a patient with choroideremia with AAV subtype 2 (AAV2) [32]. Choroideremia is an inherited disorder due to loss of the *CHM* gene and the resulting Rab Escort Protein 1 (REP-1), which leading to degeneration of the choroid and retina and blindness by the 2nd decade of life. Research moving towards clinical trials has been stymied due to a lack of an animal model with similar functional and morphological features as the human retina, since the knockout of the murine *Chm* is lethal. The authors developed a preclinical model of choroideremia using iPSCs and successfully transduced wildtype human *Chm* cDNA into these cells using AAV2 mediated therapy. They observed a functional restoration of REP-1 enzymatic activity and protein trafficking, showing that their gene therapy was successful and that iPSCs can be used as a preclinical model for choroideremia [32].

The development of genome editing tools such as zinc finger nucleases (ZFNs), transcription activator-like effector nucleases (TALENs), and the clustered regularly interspaced short palindromic repeats (CRISPR)-Cas system have facilitated gene targeting in human iPSCs [33]. These tools use double strand break induction and subsequent homology-directed repair to edit the mutations in the patients’ genomic DNA, so that the corrected gene will remain under the normal endogenous promoters and enhancers. Thus, compared to conventional viral-mediated gene replacement, gene editing using ZFNs, TALENs, or the CRISPR system can avoid genetic expression in inappropriate cell types as well as incorrect levels of expression [34].

The CRISPR-Cas system has several advantages over ZFNs and TALENs for enhancing gene targeting efficiency. Most CRISPR-Cas subtypes target DNA directly, suggesting the possibility of engineered, RNA-directed gene editing systems. This usage of easily generated RNA guides avoid the need for repeated protein design, which sets CRISPR-Cas apart from ZFNs and TALENs, which use protein-based DNA targeting motifs. Using the CRISPR-Cas9 system, Mali *et al.* targeted the endogenous AAVS1 locus in human iPSCs to achieve homology-directed repair of fibroblast-derived iPSCs [35]. Recently, Hou *et al.* developed a CRISPR-Cas system from *N. meningitides* to generate accurately targeted clones in human iPSCs with increased efficiency as compared to TALENs [36]. There several concerns with CRISPR-Cas technology in human genome editing, primarily off-target DNA cleavage [37]. However, recent experiments showed that “nickases”, or enzymes that cleave only a single strand of DNA in DNA repair, can increase the specificity and safety of the CRISPR-Cas9 system [38].

### 3.2. Gene Therapy on Patient-Specific iPSC-Derived RPE Cells

With the aim of correcting genetic defects, gene therapy has been attempted not only on patient-specific iPS cells, but also RPE cells derived from these cell lines. A proof of concept study was performed by Cereso *et al.* which used a hybrid vector comprised of AAV2 and AAV5 (AAV2/5) to mediate gene therapy to the RPE derived from iPS created from a choroideremia patient [39]. The authors successfully developed a human iPS-derived retinal cell model of choroideremia, performed gene therapy on the iPS-RPE, and showed that AAV2/5-mediated therapy could potentially restore RPE phenotype. Working with MFRP, Li *et al.* also showed that patient-specific iPS-RPE could be a recipient for gene therapy [25]. The researchers applied the AAV8 vector expressing human MFRP to iPS-RPE from patients with MFRP mutations and confirmed that gene therapy led to restoration of RPE phenotype, specifically with regards to actin organization. These studies suggest that gene therapy using AAV vectors can be applied to RPE created from patient-specific iPS for retinal diseases without previous models, and that these diseases may be potential targets for additional gene therapy trials.

### 3.3. Transplantation of iPSC-Derived RPE Cells

Considerable attention has been paid to the potential of human iPSCs as a source for regenerative medicine, disease modeling, and drug testing. In particular, the limitations in existing treatments for AMD have led to attention being given to alternative approaches in which damaged RPE is replaced by healthy RPE. In a recent landmark trial in Japan, patient specific iPSC-derived RPE cells were transplanted for the first time into a human patient with AMD. Clearance for a human trial was given after Takahashi *et al.* showed that transplantation of iPSC-derived RPE did not provoke an immune reaction nor lead to tumor growth in monkeys or mice [40]. Autologous iPSCs were created from the patient’s skin cells and then differentiated into RPE so that they would grow in a monolayer without the use of synthetic scaffolds or matrices. To achieve this, iPSC-RPE were seeded onto type I collagen gel on a Transwell insert. After the RPE reached confluence, collagenase was applied to dissolve the collagen gel and leave a sheet of RPE. A 1.3 millimeter by 3.0 millimeter cut of this sheet was then grafted into the patient’s retina following excision of her existing damaged RPE.

This marks the first clinical trial on humans using iPSCs. The safety and feasibility of using iPSCs from patients to treat their blindness is still being established, but this trial holds great potential for the advancement of translational medicine in retinal disease.

## 4. Future Directions

Patient-specific iPSCs have been shown to not only complement animal models of human disease, but also function as an excellent model in their own right. Patient-specific cell lines created from somatic cells from patients with inherited eye diseases can: (i) provide a window for testing the efficacy of gene or drug-based therapies; (ii) elucidate previously unknown mechanisms and pathways of disease; (iii) demonstrate the pathogenicity of unusual mutations in individual patients; and (iv) enable researchers to optimize parameters for successful cell replacement therapy *in vitro*. Skin-derived iPSCs can be used to investigate the function or dysfunction of a mutant gene product in tissues such as retina that are inaccessible to molecular analysis in living patients [41]. Finally, gene therapy tools such as ZFNs, TALENs, and the CRISPR-Cas system are rapidly improving the prospects of restoring the function of diseased RPE from patients with inherited retinal diseases. These patient-specific iPS-RPE, after undergoing gene therapy, can be optimized to become transplantable retinal cells, with the goal of restoring sight to patients with no other therapeutic options.

However, despite these advances, improvements still must be made in reprogramming, differentiation, and cell characterization protocols before employing this technology in clinical transplantation trials. In moving from animal models to human trials, potential safety issues must be carefully addressed. The use of potent oncogenic transgenes such as c-myc and Klf4 in the reprogramming process as outlined by Yamanaka is one area of concern [16]. If these transgenes are not silenced or are reactivated after reprogramming, genomic instability may result and not only confound results of disease modeling studies but also cause tumor formation after transplantation. To this end, iPS reprogramming protocols are still being optimized. An alternative reprogramming protocol by Yu *et al.* obviates the use of oncogenic transgenes by using a combination of Oct4, Sox2, Nanog, and Lin28 [42]. The methodology for generating iPSCs has markedly improved and now integration-free iPSCs, without transgene insertion in the host genome, can be obtained using plasmid vectors, RNA viruses, or mature microRNAs [43,44,45,46,47]. Integration-free iPSCs appear ideal since exogenous genes integrated in the host genome may affect the genetic properties of the iPSCs generated and thus modify the resulting cellular phenotypes of differentiated progeny. Additional studies are also required to ensure that the risk of rejection is significantly reduced in patient-specific iPSCs, given that immune rejection when certain tissues derived from iPSCs were transplanted into syngeneic murine hosts have been reported [26].

ESCs are still the gold standard for *in vitro* pluripotency. A significant concern of using iPSCs in development of therapies is still whether they are truly equivalent to ESCs. For example, key differences between iPSCs and ESCs in transcribed genes, epigenetic landscape, differentiation potential, mutational load, and premature senescence has been described [48]. If iPSCs cannot closely replicate ESCs, the results from studies using iPSCs must be interpreted with this in mind. Significant differences between iPSCs and ESCs may hinder the translation of study results from an *in vitro* iPSC-based disease model to human disease.

A further step likely to accelerate the integration of iPS technology in regenerative medicine is the development of industry and biotechnology collaboration in order to develop large-scale stem cell production [49]. In this way, availability of iPS-based technology will increase, making them more widespread in investigative and translational studies in the future. Patient-specific iPS-derived cells offer the hope of slowing progression or improving visual function for patients with currently untreatable retinal diseases. In addition to curing blindness, stem cell transplantation in the eye can also be seen as a model system for investigating cell-based treatments for other degenerative disorders of the CNS.

## 5. Conclusions

Stem cells have revolutionized the field of human cell culture because they provide an immortal population of pluripotent cells which can theoretically differentiate into any cell type in the body. This technology, when applied to retinal cells, has the promise to make significant contributions to our understanding of the most pressing blinding diseases of our time. Stem cells also allow for the development of therapies for exceedingly rare retinal conditions which currently have little to no funding for research. In particular, patient-specific iPSCs represent an excellent tool for modeling retinal disease since they can be generated from adult somatic cells, thus avoiding the ethical considerations involved with using embryonic stem cells. iPSCs will continue to be a sustainable method to model disease as gene therapies, drug therapies, and transplantable retinal cells continue to be developed for inherited retinal disorders.
